# The Waldegrave Interview

**Published:** 1991-09

**Authors:** 


### The Waldegrave Interview

**The Secretary of State for Health, the Rt. Hon. William Waldegrave, MP, agreed to our request for an interview, but asked that the questions should be written and he would give a written reply. The following questions were provided by members of our Editorial Committee and Mr. Waldegrave has replied to all of them.** Ed.

Q1 How will the Government measure the success or failure of the present changes to the NHS? What will be the criteria used and what methods of audit will be employed?

**A** The most important evaluation is essentially the practical one, of how the reforms work in practice. They are working well. The establishment of medical audit, the success of the fund holding scheme and the rising number of trusts, all show things are going in the right direction. Of course not everyone likes change — in the early days of the motor car some wanted red flags carried in front of them and argued that things should go slower. We shall be looking to see that the Reforms deliver a more cost-effective health service without in any way compromising the traditionally very high standards of medical care we have had in this country. Indeed we would expect the comprehensive introduction of medical audit to improve standards of care still further and I am glad that we can look to the support of the profession on this.

Achieving a more cost-effective health service means simply that we use money more wisely in the interests of patients and offer a service more responsive to their needs. This can be measured in a whole variety of ways, as for example in improvements in waiting times, better use of operating theatres and the introduction of appointment systems in out-patient departments which respect the fact that patients' time too has a value which must be respected. A wide range of evaluation studies of changes brought about by the Reforms are being conducted by the Department, by health authorities and by independent centres such as the Kings Fund. We shall take these fully into account. Equally as a politician I am in no doubt that it is the British people who will have the final say and rightly so. For all the "black propaganda" generated by my political opponents, it is heartening to see how much patients already appreciate the improvements which are coming from allowing hospitals to run themselves as NHS Trusts and from enabling their general practitioners as GP fund holders to have more control over resources. I have no doubt that as the Reforms get into full swing, the more people will understand both why change was necessary and appreciate the benefits.

We are interested in organisational performance, patient satisfaction and outcomes in terms of health.

Organisational performance can be assessed by such factors as waiting lists and staff turn-over. The fundholding scheme will, in my view, lead to a substantial reduction in the time people wait for hospital care. Staff turn-over relates to morale, and when the trusts have settled down there will be a better esprit de corps and people will like working in the organisation.

Patient satisfaction matters. Questionnaire and interview techniques are being introduced; for example a practice in Hull is assessing how patients feel about every stage in the process of referral by a GP to hospital, the hospital consultation, and the follow up by the primary health care team. Units are asking "How was your visit". So they should. Public satisfaction with public services is crucial and that is what the Patients' Charter is about.

Outcomes are ultimately of the greatest importance. We are consulting on "The Health of the Nation". Outcome management is in its infancy, but it will be a crucial issue in the years ahead. Not all hospitals achieve the same results for their patients. Equity in quality of care has never existed but by looking at variable performance and what units achieve for their patients we can improve things for everyone. Deaths from heart disease, road accidents, perinatal mortality — they all show variations. Some of this is due to factors outside the control of the NHS. But sometimes it is due to our own shortcomings.

**Q2** Teaching, University and District Hospitals all have varying degrees of teaching responsibility. This is currently carried out with difficulty because of cutbacks in university funding. Most teaching hospitals are centrally placed and therefore in an adverse position from the point of view of capital charges alone. University clinical teachers are appointed primarily to central university teaching hospitals. How can their teaching responsibility be protected in the purchaser/provider system in which patient movements and referral practices are out of their control?

**A** Whilst education is the basis of the high standards of care we wish to see, as an over-riding principle the teaching of medicine must reflect the needs of society. Students, whether nursing students or medical students, follow careers in the NHS and teaching must reflect the problems which patients present, for example the increasing age of the population and the diseases of the latter half of life. Professional education must reflect this, develop adaptability, and be based upon population centres. For example the population of central London has fallen by 5% in the last decade and the population of central London has been declining for a century. We have nine major medical schools each demanding full clinical facilities for their students. Other conurbations have this problem, and not only in the UK. More thought probably needs to be given to developing better links with the excellent district general hospitals where patients currently want to be treated. After the last war new medical schools were developed in places like Nottingham and Southampton, and others like St. Georges' relocated. One cannot protect a system if it no longer fits the broader needs of society.

**Q3** What has the implementation of this latest NHS management style cost? The bill for new notepaper alone must amount to million of pounds. There will also be increased cost for accountants and clerks to administer the charges. What future costs of this nature do you anticipate?

**A** Your question implies that additional management investment is by definition a waste of resources. I have to say I consider this to be a very short-sighted view. In many areas the NHS is simply under-managed and we need to reverse this trend since in the end it works to the detriment of clinicians and patients alike. Your reference to "accountants" and "clerks" is clearly intended to be scornful but actually we cannot hope to manage our resources better if we do not have greatly improved information on what we are doing and how much it costs. Sensible decision making on behalf of patients requires this and one has only to look at other countries of comparable prosperity to ours to see the truth of this. That said, I must equally stress that it is essential that NHS management should itself be the subject of critical scrutiny and that this is another area which needs to be kept under careful review.

Concentration on quality is central to the changes in management. Top management is becoming committed to the philosophy that quality is everyone's business. The style is to concentrate on supporting and empowering staff to improve services, and most people welcome this move away from a more traditional management style.

**Funding in 1989-90 — Around £80m**

Main areas of expenditure:

| | £ million |
|---|---|
| 100 extra consultants (funding for first four months of first 35 appointments) | 3 |
| Medical audit | 2 |
| Strengthening of finance and personnel functions and training | 17 |
| Establishment of asset registers (to prepare for introduction of capital charging) | 12 |
| Resource management initiative (RMI) | 23 |
| HISS | 5 |

**Funding for 1990-91 — Around £300m**

Main areas of expenditure:

| | £ million |
|---|---|
| 100 extra consultants | 27 |
| Medical audit | 26 |
| Quality initiatives | 10 |
| Strengthening of finance and personnel functions and training | 64 |
| RMI | 78 |
| HISS | 7.5 |
| Capital charges | 5 |
| IMTTS (Information Management Training Initiative) | 4 |
| NHS Management Communications | 4 |

**Funding for 1991-92 — Around £380m**

Main areas of expenditure:

| | £ million |
|---|---|
| 100 extra consultants* | 43 |
| Medical Audit | 49 |
| HISS | 20 |
| RMI | 87 |
| Quality | 4 |
| IMTTS | 4 |
| NHS Management Communications | 2 |

*Includes £2m for training for consultants

**Q4** Budget holders (GPs) can arrange for consultants to see their patients privately in groups at the local health centre (eg 10 dermatology cases at £30 each) at a cheaper rate than the hospital would charge (overheads, notes, transport etc). The consultant will treat this as private medicine and keep the fees. If, for example, this was done by all the dermatologists and consultants in similar low tech areas these specialities would disappear from the hospitals. Is this not ''privatisation by stealth''?

**A** You forget that the provider unit might make the arrangements to supply a locally based consultant service to a fund holder. Even if the consultant is working in his own time the result is that a more extensive service is available. What is happening is that people are looking for the most effective way of giving a high quality service. Quite often it is possible to provide a better service at the same cost — or even a lower one as in the example you give. When this is the case the money saved can be spent on other forms of NHS health care. That way everyone wins. Fundholders who spend NHS money on NHS patients are providing NHS services, whether or not that service is provided by directly employed doctors on a district pay roll or the same doctor working in a private capacity. They are providing a good service that meets peoples' needs. Why provide services in a high cost environment if it wastes money which could be better spent? Does it matter what proportion of dermatology is conducted within the walls of a hospital? Surely what matters is that patients get the care they need as fast as possible.

**Q5** If a particular unit sees fewer patients and gets less money, it will progressively wither away. What will happen to the doctors and nurses in that unit? Will they be redeployed, or be offered lower salaries or be made redundant? Conversely if a unit sees more and more patients they will need more doctors. Supposing the Manpower Committee says they cannot have them because they consider the speciality to be ''over-doctored''. Do the local managers have the power to appoint as many doctors as they can afford or will overall national policies prevail?

**A** To answer this question, one needs to know why the hospital is seeing fewer patients. If it is because general practitioners and patients consider that a higher standard of services is being provided elsewhere, then I would expect the hospital to seek to emulate their competitors and win back patients. It certainly does not follow that in these circumstances a hospital will necessarily go into irreversible decline. Equally it is nonsense to try to maintain the status quo in an organisation simply because anything else requires change in staffing. Look what happened to industries that did not modernise. We now need fewer chest physicians because tuberculosis has largely been cured but we need more orthopaedic surgeons because we can replace hip and knee joints. That is a fact of life and nobody would argue for the maintenance of sanatoria because they provided employment for doctors and nurses.

Surely everyone accepts that medical and nurse manpower should be determined by patients' needs rather than the other way round? Of course change necessarily implies that posts may disappear in one location just as new posts will be created elsewhere. That has always been the case. Doctors and nurses' skills are highly prized by the community but there must equally be a recognition by the professions that they cannot be exempt from change. Precisely how that change is brought about will depend on local circumstances and I doubt anyone would wish to see solutions imposed from Whitehall as your question rather implies.

**Q6** If a large item of capital equipment is purchased for example an MRI scanner for one million pounds, what arrangements will be made for the capital charges?

**A** All NHS capital assets over the value of £1000 are subject to capital charges. Therefore a hospital would be required to pay capital charges on an MRI scanner to its Regional Health Authority. The RHA in turn makes available this money to purchasers of health services (District Health Authorities and GP Fund Holders). We have introduced capital charges to alert health service managers and professionals that capital in the NHS is not a free good; the use of physical resources has a cost. The private sector has always realised this. Capital charges ensure fair competition exists within the NHS and between the NHS and the private sector. Capital charges should be fully reflected in the pricing of hospital services. This provides incentives for the efficient use of capital in order that a hospital remains competitive in providing a quality service at reasonable cost.

**Q7** Budget considerations may determine what treatment is available. In an individual patient the budget considerations could, for example, determine which type of pacemaker is fitted. Is this not an infringement of clinical freedom and to the detriment of patients?

**A** Budget considerations have always determined what treatment is available. It is astonishing to suggest otherwise. It is for precisely this reason that we need to have better management of NHS resources so as to ensure that money is used to meet patients' best interests. This of course does not mean that we should necessarily go for the cheapest option but it does mean that choices need to be informed by consideration of both the quality of care that will be provided and also relative cost. Failing to do this is not exercising ''clinical freedom'' but rather ''clinical irresponsibility'' since money may be wasted which could otherwise be used for the benefit of other patients.

What we need to do — and here the responsibility lies with the profession — is to determine the types of treatment that really work. Why is there such a variation in the rates of Caesarian section and hysterectomy? Why do the rates of prescription of tranquilisers vary so much. Are high rates of procedure always right? The work of Jack Wennberg at Dartmouth and Klim Macpherson at the London School of Hygiene and Tropical Medicine, suggest the answer is a resounding no. Variations are found when doctors have doubts about the effectiveness of a procedure. Let us start by being more careful about what is advised. Vogues are not always right, — think of tonsillectomy. Certainly the costs of health care are going to rise, as we can do more for people; they can also fall with more cost-effective methods like cataract surgery on a day basis. We live in the real world, and we need to spend our money to best effect. I will fight for more funds for health care — but the profession must fight to ensure that it wastes not a penny, or I will lose the battle I fight on their behalf.

Forty years on the NHS has much to be proud of. We have the best primary health care in the world. We have a more nearly fair distribution of hospital services all over the county. We have skilled and dedicated personnel. The new problems are those of the aging population, a service which like many monopolies has not treated people with the gentleness and courtesy they deserve, and which has had some of the in-built inefficiencies of monopolies. Some things needed changing. But the foundation of the NHS is secure.

